# Computational Simulation of Team Creativity: The Benefit of Member Flow

**DOI:** 10.3389/fpsyg.2019.00188

**Published:** 2019-02-07

**Authors:** Chong Zu, Hui Zeng, Xiang Zhou

**Affiliations:** ^1^Department of Social Psychology, Nankai University, Tianjin, China; ^2^School of Economics, Nankai University, Tianjin, China; ^3^Collaborative Innovation Center for China Economy, Tianjin, China

**Keywords:** team creativity, member flow, social network, computational simulation, knowledge sharing, NetLogo

## Abstract

This study simulates the team cognition model through NetLogo 6.0.2 to view a dynamic changing of team creativity during knowledge sharing when the team members perform problem-solving tasks. A hypothesis is proposed: (a) when people possess various characteristics, members who own high-level normal knowledge and have high communication frequency are suited to perform problem construction process and members who own high-level creative knowledge and have less communication frequency are suited to perform divergent exploration process; (b) member flow that old-timer is replaced by a new member, can improve the team creativity and keep it more stable. The team cognition model is based on the social network of the team, where members are assigned cognition tasks. Also, the simulation experiments are conducted in 6 conditions and each condition has one situation including “MemberFlow” procedure, and one excluding “MemberFlow” procedure. Each experiment contains 500 repetitive experiments and in each repetition, there are 100 steps of “GO” procedure are performed. The results show that the team creativity is maximal and stable in the condition of hypothesis (a), and member flow can optimize the team creativity.

## Introduction

Many influential factors of creativity, on the individual and team level, have been researched, such as openness of personality, intrinsic motivation, social characteristics, knowledge sharing, cognition processes and so on (Hennessey, 2015; Jiang et al., 2018; Lee, 2018; Xu et al., 2018). Most studies are focused on a particular factor to examine their correlations. Comparing with other methods, computational simulation can integrate several influential factors in one experiment to observe the team creativity in the environment approaching the reality. In this study, the team cognition model is simulated through NetLogo 6.0.2, using Logo language, where knowledge is shared during the team communication, and the team members generate the solutions for a specific problem through problem-solving cognitive processes. In this computational simulation, team creativity can be evaluated through how many creative solutions can be found in all solutions that can solve this problem. And the subjects in the virtual experiments are the agents, and the set of agents called “agentset.” One of the advantages that computational simulation has is that many potential characteristics can be considered in the simulation experiments, like knowledge structure and communication frequency, which are the important characters that agents possess in the simulation.

Since 1950, researchers have started to turn their interests on creativity (Guilford, 1950). After years of efforts, the essence of creativity could be understood through multi-levels, and psychologists reveal personalities, thinking modes, emotion, cognition, social characteristics can affect creativity on the individual level and organizational level, in order to provide more creative productions and better life quality. Creativity is an ability that can produce novel and useful achievements (Sternberg and Lubart, 1996). The achievements can be creative ideas, solutions for the particular problems, results from task accomplishment, and products of variety kinds of arts and et al. Team can be defined as two or more members possessing distinct characteristics and knowledge who interact dynamically, dependently and adaptively with each other to accomplish a general and valuable task, where every one is distributed specific task to perform with limited time as a member of the team (Salas, 1992). Although individual creativity of members is the foundation of the team creativity, synergistic interaction among members is crucial, so that generating creative productions are base on the individual knowledge, and knowledge sharing through communication can also affect the team creativity.

Knowledge is the base-stone of individual creativity. People cannot create new things surpass their knowledge. Therefore, when the creativity is discussed and researched, knowledge structure is a crucial characteristic in the study. In the simulation, agents need to conduct their problem-solving processes according to the knowledge pool so that their knowledge structure may affect their performance. Beyond that, many other individual characteristics, like the openness of personality, intrinsic motivation, social characteristics, can make an effect to the creativity for a creative production (Feist, 1998; Wolfradt and Pretz, 2001; Sacchetti and Tortia, 2013). These attributes can give individuals a proper intrinsic environment to generate creative achievements based on their knowledge. Besides, in terms of social characteristics, the change of social position and tie strength caused by team communication can affect the efficiency of information acquisition (Perry-Smith and Shalley, 2003; Perry-Smith, 2006). Therefore, the hypothesis has been proposed that member flow, which means in this case that using new member who is willing to communicate to ones who contains the various knowledge, would improve the team creativity and keep it more stable. In this case, member flow can be defined as that old-timer shifts out, and a new member joins this team alternatively.

All the characteristics that can affect individual creativity can be seen as a mediator between their knowledge and their performance in the cognitive processes. Thus, they are simplified in the computational simulation into efficiency variable, which represents how is the performance that people generate creative productions based on their knowledge. In the simulation, the efficiency variable is controlled as a control variable. In addition to the personal level, people collaborate with others as a team or group for a specific task through communication, which forms a social network. Simonton (2000) once suggested that successful research on creativity should place creative individuals among social network; therefore social characteristics play an important role on individual creativity, which contains the strength of the relationship, position in the social network. In terms of strength of the relationship, according to the strength-of-weak-ties theory from Granovetter (1973), weak ties interspersing among social network, which means low frequency and short time interaction and limited intimacy in the relationship, could improve on generating creative ideas. In addition, for the reason that new ideas produce from the interrelationship of previous ideas, creative ideas need an amount of information and knowledge as the basis of competence to generate creative ideas. Hence, the quality and efficiency of information acquisition are crucial. Individuals with weak ties in the social network, who can only get information less repeatedly, would get less redundant information and knowledge thereby improving the efficiency of information acquisition, compared to the ones with strong ties (Granovetter, 1973). Another social characteristic is the position including Centrality and Peripherality. Approaching the central position may increase individual creativity while surpassing a general level can impede developing creativity. That is because that people in central position would experience more relationship conflicts causing anxiety, thus strangling one’s creativity (Podolny et al., 1997). Thus, how is an individual’s strength of the relationship can be manipulated through team communication in the simulation, which is defined as the frequency of member who shares their knowledge to other members. Meanwhile, the position of the member in the team is a potential characteristic, which is an outcome of communication. In this case, this kind of communication is the knowledge sharing process.

In the individual level, the brain generates creativity, which influenced by personality, intrinsic motivation and social characteristics and other properties; in the team level, communication, and cognition compose to the brain of the team. Cooke brought about Interactive team cognition, considering that team cognition produces from the interaction among members, where people generate dynamic emergence of team cognition through interaction, negotiation, decision, and other mutual actions (Cooke et al., 2013; Cooke, 2015). Consequently, team cognition, as a complicated dynamic model, emerges from simple communication among members, which can be considered as neural connections in the brain. In many measurements, the condition of achieving tasks, producing ideas and works can be evaluated as the criteria for creativity, the processes of which need to integrate all competencies of members and knowledge, where the team communication plays a crucial role. Communication cannot only integrate productions derived from cognitive actions, but communication is also a precondition for knowledge sharing. The knowledge structure is of importance to generate new novel productions, so that knowledge diversity is one of the influence factors (Pelled et al., 1999). When the team executes cognitive activities, performs tasks, members communicate with each other for knowledge sharing to generate creative ideas and works, or solve problems, accomplish tasks. In this research, the knowledge of members is shared among the team and with the other team.

If team communication could be compared to neural connections, team cognition might be viewed as the structure of the brain. Team cognition processes in problem-solving had been extended from individual level (Reiter-Palmon et al., 2008). The problem-solving task is the research that can be solved by various methods and solutions, based on the information stored in the memory (Chiew and Wang, 2004). Many researchers developed their own models to overview this cognitive process. For instance, Bransford and Stein (1984) designed the problem-solving model as problem identification, problem representation, strategy selection, strategy application, and result evaluation. For computational simulation, an integrated team cognition model can be summarized as problem construction, divergent exploration, evaluation and conclusion. Problem construction can be valued through problem restatements, and a good problem construction can produce creative solutions with high quality (Reiter-Palmon et al., 1997; Reiter-Palmon and Robinson, 2009). In the model of Runco and Chand (1995), it is a problem finding process, aiming to decide the properties of the problem and strategy selection. In this research, agents can construct the range of the problem for subsequent divergent exploration. In the divergent exploration process, the team uses divergent thinking to explore novel and useful solutions for the problem. After exploration, the team combines scattered opinions from members to generate preliminary solutions through communication. With regard to the whole team, the solutions that generate from members independently are, inevitably, resembling or even repetitive. Therefore, in this step, integrating these solutions effectively also will be included. In the last process, evaluation and conclusion, the solutions need to be evaluated if they are useful and novel, in order to measure team creativity. In the simulation, problem construction and divergent exploration are considered and manipulated through agents, who would conduct these procedures based on their knowledge and their efficiency, which can control how many problem restatements and various solutions that they can explore. The evaluation and conclusion part can be developed in the subsequent researches.

This study builds a team cognition model to describe how the members work as a team, and then a computational simulation is programmed through NetLogo 6.0.2 and virtual experiments are performed in the BehaviorSpace in the NetLogo. Knowledge and Efficiency are the characteristics included for each team member agent, and they can share their knowledge, based on which agents execute their corresponding problem-solving processes. The aim of simulation is that a dynamic changing can be viewed in different parameters of agents and problem, and beyond that, a hypothesis can be disclosed which is member flow can improve the team creativity.

## Materials and Methods

### Team Cognition Model

The model is constructed before the simulation to conduct each of the team members what cognitive activities would proceed when they solve a specific problem and the team formation as a social network. The social network is formed from the interaction of members who are belonged to this network and then individual social characteristics are given to every member, like their tie strength and position, which may affect their performance in the team. Besides, knowledge is an important characteristic that can affect the team problem-solving results. Therefore, there are the hypotheses:

(1) When people possess various knowledge structure and communication style, members who have more normal knowledge and communicate with others more frequently, are suited to perform problem construction process; members who have more creative knowledge and communicate with others less frequently, are suited to perform divergent exploration process. This arrangement can optimize team creativity.
(2) When member flows at intervals, team creativity would be more stable and higher.

The team is constituted by two or more members who possess divergent knowledge and members who are distributed the same problem-solving task form a group: supervision group need to perform problem construction task; exploration group need to execute divergent exploration task; evaluation group is in charge of evaluation and conclusion part. In this study, the effect of evaluation and conclusion procedure to the team creativity is not considered. Meanwhile, many other properties of individuals are simplified to the efficiency that is described as a mediator element represented to how efficient members are exchanging knowledge into results of each problem-solving process. The efficiency variation as one of the individual characters is controlled as a control variable, which is affected by personality, motivation and other factors.

**FIGURE 1:**
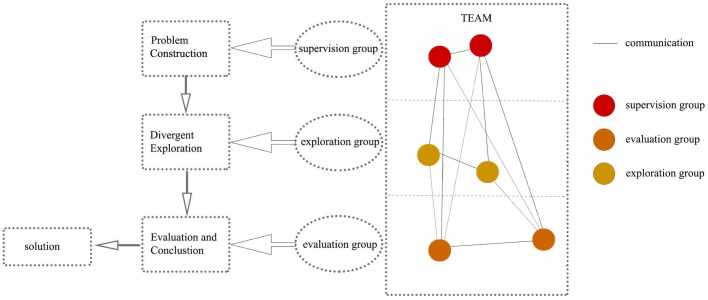
The number of members is adjustable and they communicate with each other in the team. According to the social position in the team, central members form supervision group to execute Problem Construction process, showing with red balls; Peripheral members form exploration group to generate various solutions in Divergent Exploration process, showing with yellow balls; Other members form the evaluation group to perform Evaluation and Conclusion process to integrate scattered solutions and evaluate whether final solution is creative or not, showing with orange balls.

According to Figure 1, Team Cognition Model, the team is a social network; the lines connect with other members represent the interaction, including information and knowledge sharing and normal communication. And the members separate into three groups, depending on their creativity, social characteristic and responsibility, to execute the corresponding cognitive process. While members’ social character will change along with executing cognition tasks, team creativity would change adaptively, which will conduct the computational simulation.

### Methods

**FIGURE 2:**
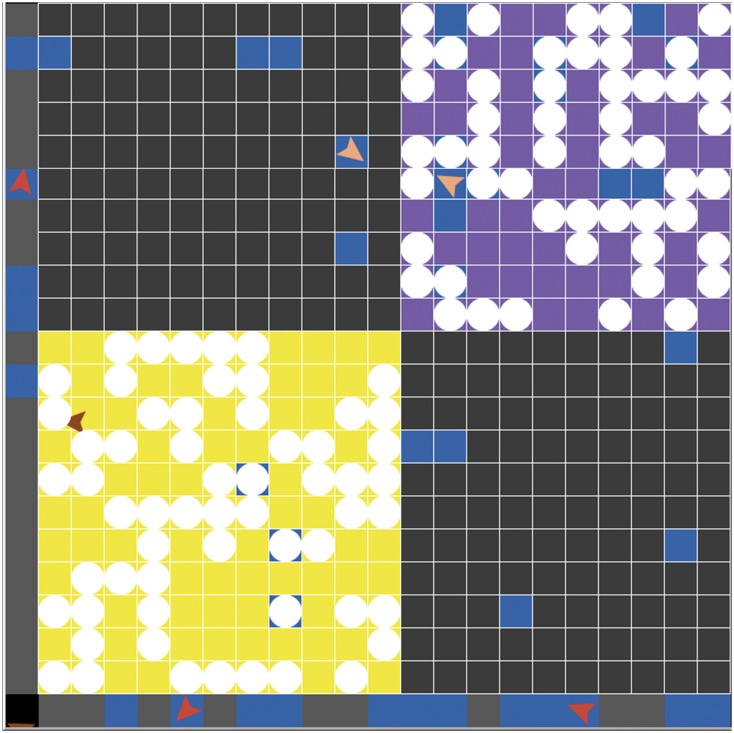
It is the “World” that can observe the member agents (triangle figures) and the rabbit agents (write round figures). The yellow part represents the normal knowledge area (normal solution area) and the purple part represents the creative knowledge area (creative solution area); the blue part represents the solutions that the team generates. When the blue part overlaps with rabbit agents, it means a feasible solution.

The simulation is implemented by NetLogo 6.0.2 (Wilensky, 1999), which is a multi-agent programmable simulation tool. The agents and their characteristics and the elements of the world can be set up in the “Setup” procedure. In the “World” (Figure 2), every agent and their behaviors can be observed. Also, their behaviors can be manipulated in the “Go” procedure. On the interface, variables can be adjusted in the certain range, and the results can be displayed through “World” and “Plot” function can show the variation of the variable at interest. Meanwhile, virtual experiments can be performed through BehaviorSpace, where the times of “Go” procedure executed in once experiment and the times of experiments can be regulated.

The psychological course is complicated and circuitous so that it is difficult to simulate all the mental processes and consider all the individual characters. Thus, some processes and characters are set up as the control variables, and in this case, they are evaluation and conclusion process and efficiency, which represents to individual characteristics that can affect the efficiency of transforming knowledge into solutions.

In the team, there are three Agentsets: Supervision Group Agentset, Exploration Group Agentset and Evaluation Group Agentsets. Based on the team cognition model that mentioned before, agents’ characteristics are knowledge structure, the frequency of communication and efficiency. Knowledge is divided into normal knowledge and creative knowledge; the ratio of them can be adjusted, and also the frequency and efficiency can be modulated. In the experiments, the characteristics of agents in the Evaluation Group Agentset and Efficiency variables of all agents are controlled at a moderate level. The problem in the simulation is a series of coordinates of rabbits who are distributed in the normal knowledge area and creative knowledge area in the “World”. The size of the “World” is based on the range of total Knowledge Pool (KP) who is a global array variable where every item of knowledge pool of all the agents are picked stochastically. The main variables are shown in Table 1.

**Table 1:** There are three Agentsets of the team members: Supervision Group Agentset, Exploration Group Agentset, Evaluation Group Agentset, and a Rabbits Agentset, whose coordinates (RCoordinate) compose the given problem need to be solved.

Agentset	Variables
Supervision Group Agentset	SKP = SKP_normal_ + SKP_creative_
	SKN_normal_
	SKN_creative_
	SC
	SE (control variable)
Exploration Group Agentset	EKP = EKP_normal_ + EKP_creative_
	EKN_normal_
	EKN_creative_
	EC
	EE (control variable)
Rabbits Agentset	RCoordinate
Global	tc
	KP = KP _normal_ + KP_creative_


*KP is the total knowledge pool, from which items are stochastically selected into SKP and EKP, which are the knowledge pool of supervision group and exploration group; KP is also decided the range of the “World”. SKN_normal_, SKN_creative_, EKN_normal_, EKN_creative_ are the variables controlled how many items are in the corresponding knowledge pool arrays. SC and EC represent the frequency of communication of members in each group. In the experiments, Evaluation Group Agentset and Efficiency variable (SE, EE) are controlled and all of their characters are set up at the moderate level. The team creativity variable (tc) is calculated in the experiment and output as the dependent variable.*

Meanwhile, the simulation is a stochastic process so that the study discards the results of the first five steps because the random numbers that are set up in the “Setup” procedure would generate the noise.

The team creativity is calculated through:

$$\text{tc}\;=\;\frac{{\text{NS}}_{\text{creative}}}{{\text{NS}}_{\text{creative}}\;+\;{\text{NS}}_{\text{normal}}}$$

In the equation, tc represents the team creativity, which means the ratio of the number of creative solutions in the total solutions the team got, where NS_creative_ is the number of creative solutions and NS_normal_ is the number of normal solutions.

The aim of simulation is to optimize the team creativity, finding a situation that can make tc maximal and stable, and the hypothesis is when members are arranged in the suitable cognition process group, and member flows at intervals, the team creativity can be maximum and stability.

## Simulation and Results

### Simulation Procedures

In the “Setup” procedure, Agentsets are bred and every variable is announced, and as well the “World” is created according to the range of total knowledge pool. All the numbers in the normal knowledge pool array compose into the normal problem area, and ones in the creative knowledge pool array compose into the creative problem area. The area formed by numbers’ combination of both knowledge pool is the redundant area, where rabbits would not generate.

Then the “Go” procedure is performed, which includes providing the problem, Knowledge sharing process, and problem construction process, divergent exploration process, and member flow process and team creativity measurement. The simulation experiments are conducted by BehaviorSpace function. In the simulation, 500 times repetitive experiments are performed under the same condition of the experiment (A, B, C, D, E, F) and in each repetition 100 steps of “Go” procedures are conducted. For optimizing the team creativity, 6 pair experiments are conducted, and each pair of experiment containing “MemberFlow” procedure and excluding “MemberFlow” procedure, which are A/a, B/b, C/c, D/d, E/e, F/f (e. g., A experiment contains “MemberFlow”, an experiment excludes “MemberFlow”). The specific setup can be seen in Table 2 and the Supplementary Data Sheets S1–S12 are the output files from BehaviorSpace experiments (1 and 2 for A/a, 3 and 4 for B/b, 5 and 6 for C/c, 7 and 8 for D/d, 9 and 10 for E/e, 11 and 12 for F/f), which are analyzed in the Results section.

**Table 2:** A/a, B/b, C/c, D/d, E/e, F/f are 6 pair of experiments.

Vabiables	A	a	B	b	C	C	D	d	E	e	F	f
SKN _normal_	8	8	5	5	8	8	2	2	8	8	8	8
[1, 10]												
SKN_creative_	2	2	5	5	2	2	8	8	2	2	2	2
[1, 10]												
SC	10	10	5	5	2	2	10	10	2	2	10	10
[1, 10]												
SE (CV)	10	10	10	10	10	10	10	10	10	10	10	10
[1, 20]												
EKN_normal_	2	2	5	5	2	2	8	8	8	8	8	8
[1, 10]												
EKN_creative_	8	8	5	5	8	8	2	2	2	2	2	2
[1, 10]												
EC	2	2	5	5	10	10	2	2	10	10	2	2
[1, 10]												
EE (CV)	10	10	10	10	10	10	10	10	10	10	10	10
[1, 20]												
MemberFlow	on	off	on	off	on	off	on	off	on	off	on	off
On/Off												


*The range of SKN_normal_ is [1, 10] so that 8 is a high level of number of items in the knowledge pool array. CV means control variable.*

### Results

In terms of all experiments, the initial knowledge pool arrays of members are limited in 10 items, the ratio of the size of normal knowledge and creative knowledge can be adjusted through variable SKN_normal_, SKN_creative_, EKN_normal_, EKN_creative_. Thus, in the A/a experiments, agents in the supervision group agentset own the high level of normal knowledge and high level of communication frequency; on the contrary, agents in the exploration group agentset possess the high level of creative knowledge and low level of communication frequency. The variables in the experiments B/b are set up at the moderate level. Comparing with A/a experiments, the C/c experiments have the different setup in communication frequency; D/d experiments have the different setup in members’ knowledge structure. The E/e and F/f experiments perform with the low creative knowledge level of all supervision and exploration group agents and with different setup of communication frequency.

The result of team creativity (tc) is collected and analyzed, which is shown in Table 3. In once experiment, each step can generate a value of tc, so that tc_mean_ represents to the general team creativity of this experiment, which is the ratio of creative solutions among all solutions so that the range of tc is [0, 1]. When tc > 0.5 means that the virtual team generates more creative solutions and less normal solutions. In the study, a high probability of the mean of team creativity who reaches to 0.8 means that most repetitions of the experiment in certain condition can get a mean above 0.8 in 100 steps, which represents as *P*_mean_. *P*_0.95_ means the probability of repetitive experiments whose values of team creativity in 95% steps are above a certain value so that higher probability means a more stable team in these repetitions of the experiments. *P*_mean_ shows the team stability in the experiment, and *P*_0.95_ shows the team stability in all the repetitions of the experiments.

**Table 3:** tc represents to team creativity, whose range is [0, 1]; tc_mean_ represents to the mean of team creativity in once repetition of each condition of experiments.

	tc ≧ 0.8		tc ≧ 0.7		tc ≧ 0.6		tc ≧ 0.5		tc ≧ 0.4		tc ≧ 0.3		tc_mean_
	*P*_mean_	*P*_0.95_	*P*_mean_	*P*_0.95_	*P*_mean_	*P*_0.95_	*P*_mean_	*P*_0.95_	*P*_mean_	*P*_0.95_	*P*_mean_	*P*_0.95_	
A	0.664	0.398	0.968	0.846	0.998	0.976	1.000	0.996	1.000	1.000	1.000	1.000	0.820
a	0.656	0.364	0.950	0.832	0.996	0.972	1.000	0.974	1.000	1.000	1.000	1.000	0.817
B	0.090	0.012	0.482	0.252	0.886	0.630	0.984	0.908	1.000	0.976	1.000	1.000	0.693
b	0.082	0.030	0.526	0.248	0.850	0.648	0.986	0.908	1.000	0.988	1.000	1.000	0.694
C	0.000	0.000	0.074	0.024	0.443	0.158	0.742	0.460	0.944	0.760	0.998	0.940	0.559
c	0.000	0.000	0.044	0.010	0.344	0.112	0.722	0.440	0.942	0.740	0.994	0.918	0.553
D	0.000	0.000	0.032	0.002	0.232	0.080	0.666	0.346	0.934	0.686	0.994	0.916	0.535
d	0.002	0.000	0.022	0.006	0.234	0.088	0.604	0.344	0.902	0.672	0.994	0.910	0.528
E	0.000	0.000	0.000	0.000	0.000	0.000	0.012	0.002	0.096	0.026	0.386	0.116	0.283
e	0.000	0.000	0.000	0.000	0.000	0.000	0.020	0.002	0.108	0.022	0.372	0.114	0.280
F	0.000	0.000	0.000	0.000	0.002	0.000	0.010	0.002	0.076	0.012	0.320	0.090	0.272
f	0.000	0.000	0.000	0.000	0.000	0.000	0.008	0.000	0.076	0.012	0.300	0.082	0.267


*P_mean_ means the probability of the mean of team creativity who reaches to the certain value. P_0.95_ means that the probability of repetitive experiments whose values of team creativity in 95% steps are above a certain value.*

### Individual Knowledge Structure

**FIGURE 3:**
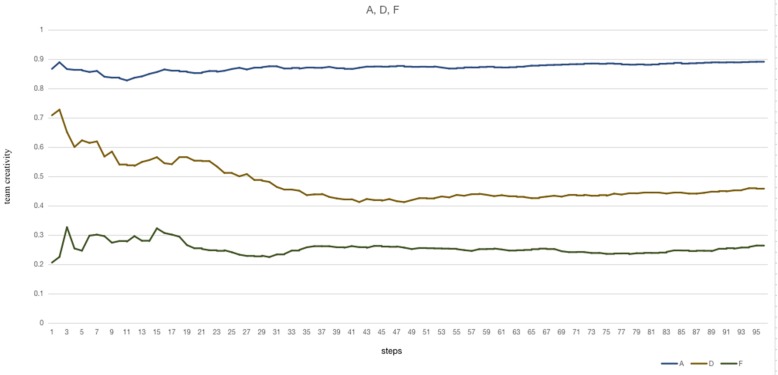
The variations of team creativity in the A, D, F experiment are illustrated.

In the F, D, A experiments (Figure 3), the communication frequency of both supervision group and exploration group are the same. There are both low level of creative knowledge in both groups in the F experiment, whose mean of team creativity (tc_mean_ = 0.272) of the whole experiment is lowest; in the D experiment, members in the supervision group own high level of creative knowledge, and on the contrary, the exploration group own low level of creative knowledge, whose mean of team creativity is 0.559; a high value of tc_mean_ (0.820) shows in experiment A, whose members in the supervision group own relatively low level of creative knowledge and members in the exploration group possess the high level of it. Also, in the A experiment, most repetitions and steps in the same repetition generate a high value of team creativity, and most of the time the team creates more normal solutions in the F experiment.

Therefore, members who own a high level of creative knowledge are suited in the exploration group and ones who own a high level of normal knowledge are suited in the supervision group. This arrangement can optimize team creativity.

### Communication Frequency

**FIGURE 4:**
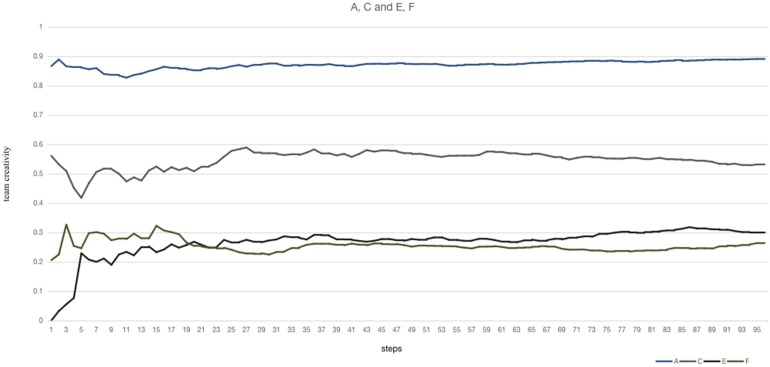
The variations of team creativity in the A, C and E, F experiment are illustrated.

When the members are arranged according to their knowledge structure becomingly, such as the A, C experiment. While in the C experiment, members in the supervision group have a low level of communication frequency and members in the exploration group are willing to communicate with others, which leads to a relatively low mean of team creativity (tc_mean_ = 0.559) and nearly 30% of repetitive experiments generate an average of team creativity under 0.5, which means more normal solutions, in the A experiment, members in the exploration group have a low level of communication frequency and members in the supervision group are more willing to communicate with other ones, which leads to a relatively high tc_mean_ (0.820) and, in most repetitive experiments and steps in once repetition, team creativity can reach above 0.7 (*P*_mean_ = 0.968, *P*_0.95_ = 0.846) (Figure 4).

In the condition that all members of the team own low level of creative knowledge, when members in the supervision group are more willing to communicate, as the performance in the F experiment, tc_mean_ (0.272) is slightly less than the mean of team creativity in the E experiment whose members in the exploration group are more willing to communicate.

Therefore, members who have a high level of communication frequency are suited in the supervision group and members who have a low level of communication frequency are suited in the exploration group. This arrangement can optimize team creativity.

### Member Flow

**FIGURE 5:**
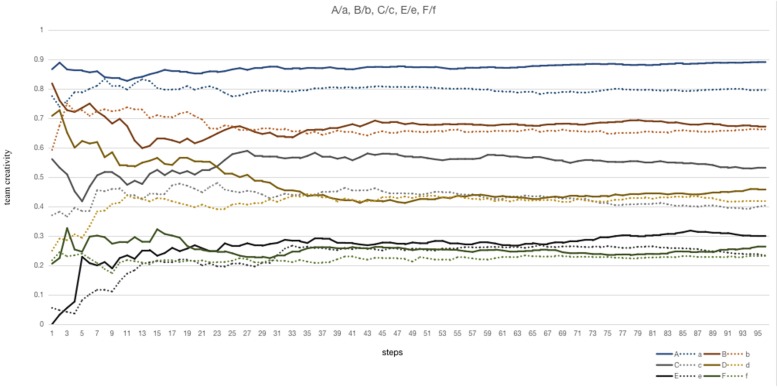
The variations of team creativity in the A/a, B/b, C/c, D/d. E/e, F/f experiment are illustrated.

Comparing to the A, C, D, E, F experiment, the different variable is that “MemberFlow” procedure is excluded in the a, c, d, e, f experiments (Figure 5), and the result of tc_mean_ are lower than the corresponding experiments who include “MemberFlow” procedure. To the *P*_mean_ and *P*_0.95_, the probability of team creativity in the high-level range is higher in the experiments who include “MemberFlow” procedure, such as A, C, D, E, F experiment. However, the improvement is unobserved in the B/b experiment, so that the member flow moderating effect is higher when characteristics of members are divergent.

Therefore, member flow has a positive moderating effect when the team wants to optimize their team creativity.

## Discussion and Conclusion

The results display that the member arrangement proposed in the hypothesis can optimize team creativity and member flow can moderate the team creativity in most situation. Many previous pieces of research show that changing membership can improve team creativity. Rotating randomly a subset of group members when they performed divergent exploration tasks can enhance group creativity (Choi and Thompson, 2005). Social identity is one reason for the members of an original group to accept rotating member from a different place, and which can increase knowledge stock of this group, hence enhancing their performance (Kane et al., 2005). In accordance with other laboratory studies, the result from the computational simulation is reasonable. In terms of transactive memory system (TMS) of the group, partial membership change creates inefficient TMS processes because TMS structure that new members rely on is developed by old-timers in their original group (Lewis et al., 2007), and anticipating of membership change can make transactive memory more difficult to build (Blanchet and Michinov, 2017). Consequently, in the future, transactive memory system and cognitive conflict should be considered during simulation for a more comprehensive study about member flow, except for the knowledge structure and communication frequency of members in the team. In addition to member flow, many other adaptive changing can affect team creativity that researchers cannot examine through traditional laboratory experiments or questionnaires. The computational simulation may be considered as another method to explore team variations through problem-solving processes or other cognition processes. Moreover, people in the peripheral position, who possess more external interaction with other social networks, have a higher level of creativity; correspondingly, people in the central position, who possess less external interaction, also can develop a higher level of creativity (Perry-Smith and Shalley, 2003; Perry-Smith, 2006). Thus, external knowledge sharing can be considered in the next step to simulate. Besides, the evaluation group and efficiency variable are controlled in this study, which can be considered in the following researches.

In addition to many other factors that can affect the team creativity, also the creativity has been researched in various methods and developed various interpretations. In terms of neuroimaging method, insight, as a kind of creative cognition, which is defined as a process that people can solve a problem from the state of not knowing to knowing abruptly, and the ‘Aha!’ experiences occurred in insight problem-solving have a high positive correlation with positive affect and fluent cognition which can improve individual creative thinking (Shen et al., 2016). This insight process also has been examined through this method and many relative studies have found consistently that anterior cingulate cortex and prefrontal areas are related to insight (Dietrich and Kanso, 2010) and right hemispheric dominance theory of creative thinking also applies to creative insight (Shen et al., 2013). Therefore, if the structure of the team and the interaction among members could be considered as the brain that may construct the team creativity and then generate creative achievements efficiently, the insight problem-solving process may be possible to be found in the team creative problem-solving process, which can be considered in the next stage of computational simulation. Besides and Dietrich (2018) proposed a new theoretical framework of creativity to separate this concept into three modes, and among them the flow mode concept can be made a analogy with the member flow in the team.

In conclusion, the preponderance of computational simulation can be seen in this study. This method can integrate all contents of researches that psychologists concern, to observe variations of team creativity qualitatively through simulating a series of cognition processes and considering various individual characteristics instead of focusing on one of them, although this method cannot obtain a sufficient external validity like other laboratory experiments.

In terms of the process of simulation, before which a team cognition model has been set up including members’ responsibility distribution: supervision group, exploration group, evaluation group; besides agent behaviors are included: knowledge sharing and the problem solving cognitive processes: problem construction, divergent exploration, evaluation and conclusion. According to previous researches, knowledge structure and social characteristics can affect the team creativity, so that these properties are put into the simulation; other individual characteristics are also important but they are controlled in the simulation experiments as efficiency variable (SE, EE). Then, the team cognition model is simulated through NetLogo 6.0.2. The results show that knowledge structure and communication frequency can affect the team creativity and when people gain the various characters in both, a suitable arrangement can optimize the team creativity and it can be more stable and higher when member flows.

## Author Contributions

CZ designed this study, collected and analyzed the data with the assistance of XZ, and wrote the manuscript. XZ and HZ provided the critical emendations.

## Conflict of Interest Statement

The authors declare that the research was conducted in the absence of any commercial or financial relationships that could be construed as a potential conflict of interest.

## Supplementary Material

DATA SHEETS S1, S2 The outputs of A/a experiments through BehaviorSpace function.

DATA SHEETS S3, S4 The outputs of B/b experiments through BehaviorSpace function.

DATA SHEETS S5, S6 The outputs of C/c experiments through BehaviorSpace function.

DATA SHEETS S7, S8 The outputs of D/d experiments through BehaviorSpace function.

DATA SHEETS S9, S10 The outputs of E/e experiments through BehaviorSpace function.

DATA SHEETS S11, S12 The outputs of F/f experiments through BehaviorSpace function.
